# Versatile Tool for Droplet Generation in Standard Reaction Tubes by Centrifugal Step Emulsification

**DOI:** 10.3390/molecules25081914

**Published:** 2020-04-21

**Authors:** Martin Schulz, Sophia Probst, Silvia Calabrese, Ana R. Homann, Nadine Borst, Marian Weiss, Felix von Stetten, Roland Zengerle, Nils Paust

**Affiliations:** 1 Hahn-Schickard, Georges-Koehler-Allee 103, 79110 Freiburg, Germany; 2 Laboratory for MEMS Applications, IMTEK—Department of Microsystems Engineering, University of Freiburg, Georges-Koehler-Allee 103, 79110 Freiburg, Germany

**Keywords:** microfluidics, centrifugal step emulsification, droplet generation, emulsification, compartmentation, digital amplification, standard devices, digital droplet polymerase chain reaction (ddPCR), digital droplet loop-mediated isothermal amplification (ddLAMP)

## Abstract

We present a versatile tool for the generation of monodisperse water-in-fluorinated-oil droplets in standard reaction tubes by centrifugal step emulsification. The microfluidic cartridge is designed as an insert into a standard 2 mL reaction tube and can be processed in standard laboratory centrifuges. It allows for droplet generation and subsequent transfer for any downstream analysis or further use, does not need any specialized device, and manufacturing is simple because it consists of two parts only: A structured substrate and a sealing foil. The design of the structured substrate is compatible to injection molding to allow manufacturing at large scale. Droplets are generated in fluorinated oil and collected in the reaction tube for subsequent analysis. For sample sizes up to 100 µL with a viscosity range of 1 mPa·s–4 mPa·s, we demonstrate stable droplet generation and transfer of more than 6 × 10^5^ monodisperse droplets (droplet diameter 66 µm ± 3 µm, CV ≤ 4%) in less than 10 min. With two application examples, a digital droplet polymerase chain reaction (ddPCR) and digital droplet loop mediated isothermal amplification (ddLAMP), we demonstrate the compatibility of the droplet production for two main amplification techniques. Both applications show a high degree of linearity (ddPCR: R^2^ ≥ 0.994; ddLAMP: R^2^ ≥ 0.998), which demonstrates that the cartridge and the droplet generation method do not compromise assay performance.

## 1. Introduction

Water-in-oil (W/O) droplets are widely used for the generation of micro-compartments that find application in various biochemical reactions. The fields of application range from DNA and RNA analysis [1,2,3,4] to applications in the chemical-, medical-, cosmetic-, or food-industry [5,6,7,8]. For the processing of monodisperse droplets, the existing microfluidic technologies can be classified into two general categories: (i) On-chip systems, where the droplets remain in the cartridge and analysis and droplet manipulation are performed on-chip [9,10,11,12,13,14,15,16]. (ii) Off-chip systems, where the droplets are transferred into a reaction tube and downstream analysis and/or further manipulation steps are performed in bulk [4,17,18,19,20]. From an application point of view, devices of the first category offer an ideal method for direct quantification of digital assays but require the use of specialized equipment. This is different for off-chip systems, where the droplets are transferred into an external container (e.g., reaction tube) after droplet generation. The transfer into a collecting tube allows further handling in standard laboratory devices. Depending on the application needs, the droplets can be incubated, cycled and transferred to other devices for visualization [21], droplet sorting [22,23] or droplet-fusion [24].

For off-chip solutions, many different droplet generation systems exist [4,17,18,19,20]. However, most of them are difficult to manufacture, require complex experimental set-ups or need mineral oil for droplet generation [17,18,19]. The implementation of centrifugal microfluidics [25] enabled droplet generation on standard centrifuges without the need for any external pressure pumps, tubings, fittings or specialized devices, as it is the case for pressure driven systems [4,20]. But the so far developed systems [17,18,19] use mineral oil for droplet generation which has unfavorable properties compared to fluorinated oil, which is preferred for biochemical assays due to its high biocompatibility [26]. The low solubility of organic molecules in fluorinated oils restrict cross-talk between the droplets [27] and the low cohesive energy leads to a low value of the solubility parameter, which results in a high solubility of oxygen and carbon dioxide [28], a key factor for cell survival and culturing of singularized cells in aqueous droplets [29]. Besides these advantages, mineral oils is commonly used in centrifugal microfluidics due to its convenience in the process of droplet generation. The lower density of the mineral oil (~800 Kg·m^−3^ [30]) cause the aqueous droplets (~1000 Kg·m^−3^ [30]) to descend in the oil phase, therefore, the centrifugal force automatically propels the droplets radially outwards. In contrast, the higher density of fluorinated oil (~1600 Kg·m^−3^ [30]) results in ascending droplets, which requires a more sophisticated fluidic design for droplet transfer.

In this work, we present an off-chip system that uses centrifugal step emulsification [12,16] to generate aqueous droplets in fluorinated oil. The cartridge is manufactured out of two parts, one monolithic fluidic layer and a sealing foil and can be processed in 2 mL reaction tubes on standard centrifuges. The only remaining manual handling steps are to load the oil and reaction mix, start the centrifuge and to dispose the cartridge after the emulsification. The emulsion remains in the reaction tube and is then ready for further handling.

Stable droplet generation and transfer is demonstrated for common sample viscosities between 1 mPa·s to 4 mPa·s. Biochemical compatibility of the cartridge is demonstrated with two exemplary amplification reactions, a digital droplet polymerase chain reaction (ddPCR) and a digital droplet loop-mediated isothermal amplification (ddLAMP) reaction.

## 2. Workflow and Microfluidic Cartridge Design

**Figure 1:**
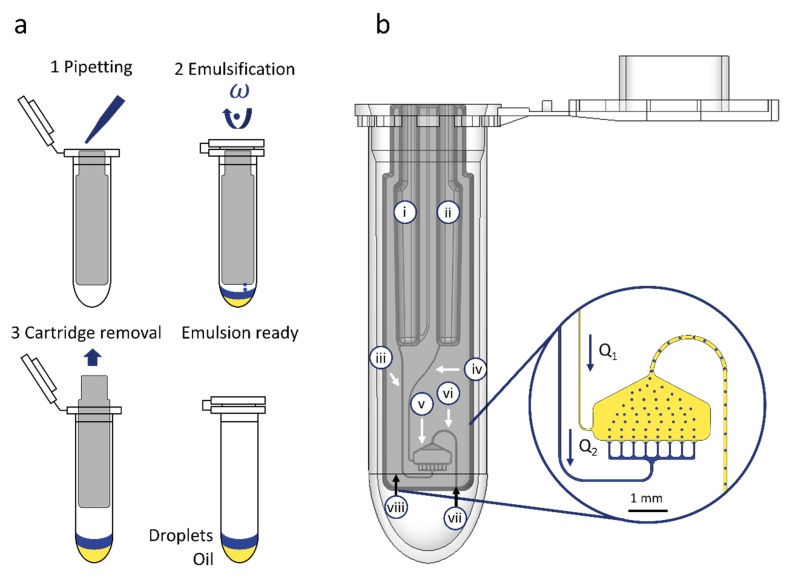
Overview of workflow and microfluidic cartridge design. (**a**) Schematic depiction of the workflow for cartridge loading by pipetting oil and water-based sample, emulsification and cartridge removal. (**b**) 3D-CAD view of the cartridge in a standard 2 mL tube with an insert depicting the droplet generation and transfer into the tube: (i) Sample inlet; (ii) oil inlet; (iii) sample supply channel; (iv) oil supply channel; (v) droplet generation unit; (vi) outlet channel; and (vii) outlet nozzle where the droplets exit the cartridge. (viii) rcf reference point. In both cases, the blue color represents the droplets and yellow color the fluorinated oil.

The cartridge has a size of 33.6 × 7.8 × 3.4 mm^3^ (h·w·d), is designed to fit into a standard 2 mL centrifuge tube and consists of two parts: a monolithic fluidic layer and an adhesive cover foil (for detailed description see Section 4.1). The workflow for emulsification can be described as follows (see Figure 1a): The cartridge is placed into a 2 mL reaction tube and both fluorinated oil and the aqueous sample is pipetted into the particular inlets. After closing the lid to prevent evaporation and contamination, the tube is inserted into a standard laboratory centrifuge for droplet generation by centrifugal step emulsification [14,16]. After processing at a fixed centrifugal acceleration of 80 g (calculated for the rcf reference point; see Figure 1b (viii)), the cartridge is removed from the tube and the generated emulsion is ready for follow-up steps such as incubation or, transfer to other devices.

The fluidic layer of the cartridge houses six microfluidic elements (see Figure 1b): Inlets for the oil (i) and the sample (ii), two supply channels for sample supply (iii) and fluorinated oil supply (iv), one droplet generation unit (DGU) (v) and an outlet channel (vi) for transporting the droplets to an outlet nozzle (vii) and out of the cartridge into the tube. Both inlets are designed to take up to 100 µl of sample and oil, each. The supply channels are designed with a defined cross section to control the flowrate of the oil (Q_1_) and sample (Q_2_). One crucial requirement for droplet transfer out of the cartridge into the tube, is an oil co-flow Q_1_ to transport the droplets through the outlet channel. In the present cartridge, the maintenance of this oil co-flow is ensured by fluidic design. Both inlets (i and ii) are designed with the same hydrostatic height and are supplied with the same volume. Since the oil has a higher density, the propelling centrifugal pressure is higher for the oil phase. To realize a slower transfer of the oil anyhow, the supply channel for oil supply (iv) is equipped with a higher fluidic resistance than the sample supply channel (ii), as long as the viscosity of the sample remains ≥ 4 mPa·s. Details of the fluidic design calculations and channel and chamber dimensions are listed in ESI 2, S1.

The droplet generation unit is designed with an array of 8 nozzles and manufactured with a mean cross section of 50 ± 2.63 × 22 ± 0.69 µm^2^ (variance between individual nozzles due to manufacturing) in order to allow the generation of droplets with a diameter of about 66 µm (for detailed droplet diameter evaluation see Section 3.1). The outlet channel is designed as an inverse siphon and manufactured with a cross section of 100 × 100 µm^2^ to fit a single droplet at a time which is transported through the channel out of the cartridge into the tube. The system can be easily adapted for the generation of smaller or larger droplets by adjusting the geometry of the nozzle cross section as published by Schuler et al. [14].

## 3. Results and Discussion

### 3.1. Droplet Formation and Transfer

Droplet formation and transfer has been tested with different glycerol–water mixtures to mimic a common range of sample viscosities between of 1–4 mPa·s. In all cases, 100 µL sample was emulsified in Novec 7500 with 5% picosurf-1. First, the fluidic performance of the cartridge was evaluated by imaging the droplet generation and the droplet transfer using a stroboscopic set-up. The set-up allows the precise control of the rotational frequency as well as the observation of the moving liquids by the stroboscopic camera setup. In addition to the live observation, high resolution images can be recorded and analyzed afterwards. In a second step, droplet generation into a reaction tube was performed using a standard laboratory centrifuge with the option to extract the droplets after generation for further analysis. In all experiments, droplets were generated with a fixed centrifugal acceleration of 80 g (calculated at the rcf reference point; see Figure 1).

**Figure 2:**
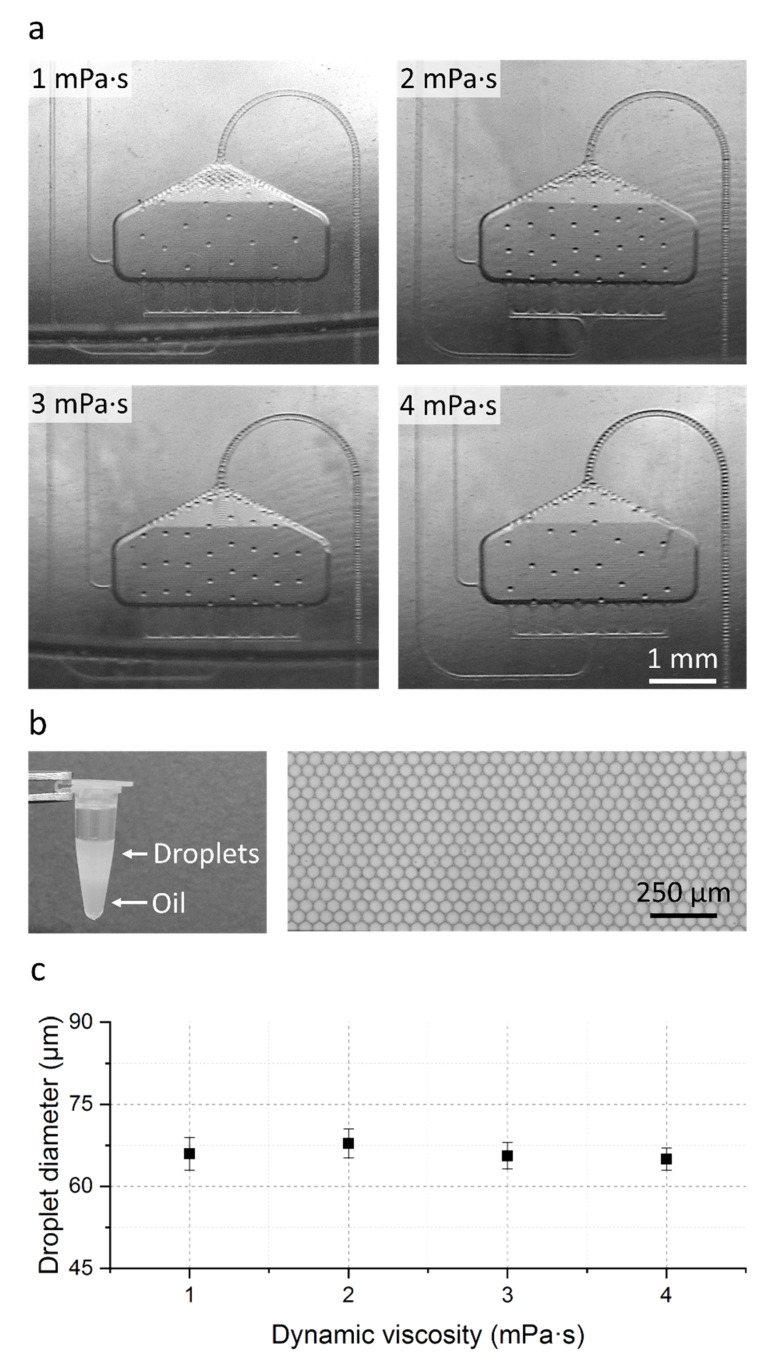
Results of the fluidic evaluation. (**a**) Stroboscopic images of the droplet generation and transfer process for all tested viscosity cases. (**b**) Picture of the generated emulsion in a tube (left); Microscopic image of the generated droplets in a droplet monolayer after transfer to a counting chamber (right, cropped image section of a representative picture). (**c**) Measured droplet diameter in relation to the sample viscosity for all tested viscosity cases. For each viscosity case, two cartridges were analyzed. Droplet diameter was measured with an automated ImageJ script (*n* = 1000, for details see ESI 2, S2). Error bars represent the standard deviation.

As shown in the stroboscopic images (see Figure 2a) and in the stroboscopic video in the electronic supplementary information ESI 1, stable droplet generation could be observed for all tested viscosity cases. Droplet generation rates up to 1160 droplets·s^−1^ are reached (see Table 1) which enables the presented cartridge to emulsify 100 µL sample into 6.65 × 10^5^ monodisperse droplets in less than 10 min.

Also visible in Figure 2a is a droplet accumulation at the top of the droplet generation area with decreasing sample viscosity. This droplet accumulation originates from the increasing sample flow rate Q_2_ with respect to the constant oil flow rate Q_1_ and is highest for the 1 mPa·s case (see Table 1).

In order to investigate if this droplet accumulation leads to droplet merging in the DGU or during droplet transfer, the in-tube generated droplets were extracted from the reaction tube, transferred into an observation chamber and evaluated using a bright field microscope. Measurements of the droplet diameter with an automated ImageJ script (*n* = 1000, see ESI 2, S2) revealed a mean diameter of 66 µm ± 3 µm with a low CV in droplet diameter of ≤4% (see Figure 2b,c). With this we could demonstrate that the droplets remain intact during the transfer and that even for the lowest viscosity with the highest accumulation in the DGU no droplet merging occurs making the cartridge usable for a variety of different samples.

### 3.2. Application Examples: ddPCR and ddLAMP

In addition to the evaluation of the fluidic performance, the biochemical compatibility of the cartridge was tested by applying two existing and well established assays on the system: A ddPCR targeting the human cystic fibrosis transmembrane conductance regulator (CFTR) gene and a ddLAMP targeting the enniatin synthetase gene of *Fusarium poae*.

In both application examples, reaction mixes containing the amplification reagents and template DNA were emulsified in the cartridge as described in Section 3.1. After droplet generation, the generated emulsions were transferred into PCR tubes, incubated in a standard thermo-cycler and finally transferred to a standard counting chamber for fluorescent readout using an automated fluorescence microscope. Total number of droplets and positive droplets were counted by the automatic image recognition software of the microscope and the resulting concentration was calculated using Poisson statistics (see ESI 2, S4). The following samples were tested: a ten-fold serial dilution of a quantified DNA sample for the ddPCR (see Section 4.5) and a ten-fold serial dilution of an unknown DNA sample for the ddLAMP (see Section 4.6).

**Figure 3:**
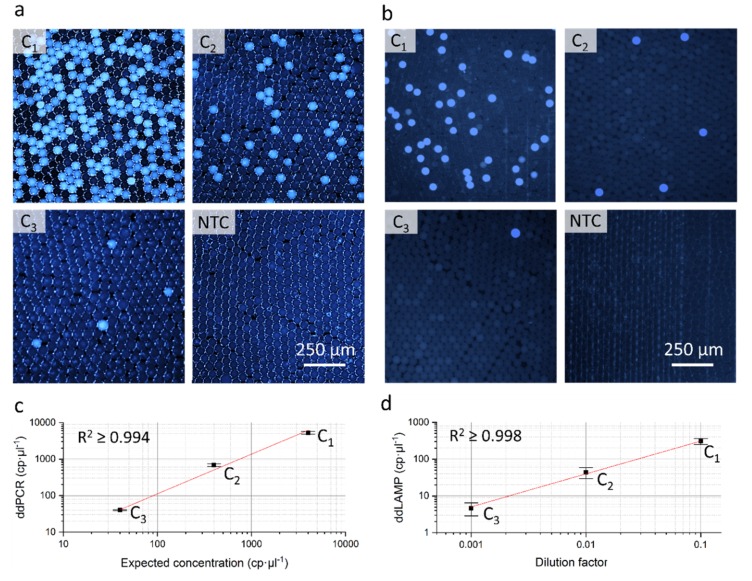
Results of the application examples digital droplet polymerase chain reaction (ddPCR) and digital droplet loop mediated isothermal amplification (ddLAMP). Fluorescent microscopy images of the droplet mono layers in a readout chip for the tested concentrations (C1–C3) and the non-template control (NTC) for the ddPCR (**a**) and the ddLAMP (**b**) (representative image sections cropped). Bright blue droplets represent positive droplets. Comparison of determined and expected concentrations in case of the ddPCR (**c**). Measured concentration in relation to the dilution factor of the unknown sample for the ddLAMP (**d**). Error bars represent the standard deviations of three independent runs, the red line the linear fit curve. RAW data is shown in ESI 2, S5.

As visible on the fluorescent microscopy images of the droplet mono layers of the ddPCR (see Figure 3a) and the ddLAMP (see Figure 3b) a clear differentiation between negative and positive droplets is possible. Furthermore, the measured template concentrations of both dilution rows show a high degree of linearity (ddPCR: R^2^ ≥ 0.994; ddLAMP: R^2^ ≥ 0.998) and in case of the ddPCR a good concordance to the expected DNA concentrations. This demonstrates that the cartridge does not compromise the performance of the two assays.

## 4. Materials and Methods

### 4.1. Microfluidic Cartridge Design and Manufacturing

The cartridge was designed using network simulation-based CAD modeling [31] (Dassault Systèmes SE, SolidWorks, Vélizy-Villacoublay, France; MathWorks Corp., Matlab, Natick, MA, USA). The cartridge was manufactured at the Hahn-Schickard Lab-on-Chip foundry [32] with the following steps:

(1) Manufacturing of a master mold by micromachining (KERN Evo mill, KERN Microtechnik GmbH, Eschenlohe, Germany) in PMMA (Evonik AG, Essen, Germany). Followed by a quality check of the manufactured structures using a confocal microscope (DUO Vario, Confovis GmbH, Jena, Germany).
(2) Manufacturing of a positive molding tool in Polydimethylsiloxan (PDMS) by pouring a mixture of Elastosil 607 and Elastosil 675 (1:1, with monomer/crosslinker ratios of 9:1 and 1:1 respectively) into the milled substrate and applying centrifugal force (Zentrifuge Rotanta 460 R, Hettich GmbH, Kirchlengern, Germany).
(3) Replication by hot embossing using the manufactured molding tool in cyclo olefin-copolymere (COC) (TOPAS COC 5013, TOPAS Advanced Polymers GmbH, Raunheim, Germany).
(4) Manual sealing of the cartridge using a pressure sensitive adhesive film (9795R diagnostic tape, 3M Corp., Saint Paul, MN, USA).

No coating or surface treatment was applied to the cartridges. During droplet generation the DGU is wetted with fluorinated oil which forms a thin film between the channel walls and the dispersed phase (sample) which prevents water adhesion at the channel walls.

### 4.2. Centrifugal Actuation

Throughout this work, two configurations for centrifugal actuation were used:

(a) For observation of droplet generation and transfer, a programmable centrifuge (LabDisk-Player 1st generation, custom manufactured by QIAGEN Lake Constance GmbH, Stockach, Germany) with a modification for stroboscopic imaging (BioFluidix GmbH, Freiburg im Breisgau, Germany) were used.
(b) For in-tube droplet generation and collection, a standard laboratory centrifuge (Cfuge 3, LLG GmbH, Meckenheim, Germany) was used.

### 4.3. Droplet Generation

Throughout all experiments, fluorinated oil (HFE, Novec 7500 3M Corp., with the addition of an interface stabilization agent Pico-Surf 1 5%, Dolomite Ltd., Royston, UK; material parameters see ESI 2, S1) was used. Pico-Surf 1 has proven to have a strong stabilizing effect on droplet stability regardless of the aqueous phase. This has been demonstrated for different assay types where differing surfactant concentrations of 2%–5% were used [13,14,15]. For the PCR however a concentration of 5% was necessary to ensure droplet stability during cycling induced stress. As the presented cartridge is intended to be used for a variety of biochemical applications a surfactant concentration of 5% was chosen to cover isothermal- as well as PCR assays.

Pipetting was performed by the use of Research Plus/Reference 2 pipettes (Eppendorf AG, Hamburg, Germany) and gel loading pipette tips (Corning Life Science Inc., New York City, NY, USA). The used pipette tips allow to reach down to the inlet chamber bottom during pipetting, to prevent an air bubble entrapment during inlet filling.

### 4.4. Water Glycerol Mixture Preperation

The viscosities of the glycerol water mixtures were calculated by following the density model developed by Volk et al. [33,34]. Mixtures were prepared by mixing defined volumes of 99.5% p.a. Glycerol (3783.1 Rotipuran, Carl Roth GmbH, Karlsruhe, Germany) with distilled water (UltraPure Ndase/Rnase free 10977-035, Invitrogen Corp., Carlsbad, CA, USA). The used mixtures are listed in ESI 2, S1.

### 4.5. ddPCR

The ddPCR reaction mix was prepared using following final concentrations: 1× ddPCR Supermix for probes (No dUTP) (Bio-Rad Laboratories Inc., Hercules, CA, USA), 900 nM primers, 250 nM probe and 4000 nM Alexa 647-fluorophor (Thermo Fisher Scientific Inc., Waltham, MA, USA) for improved recognition of negative droplets. gBlock DNA (Integrated DNA Technologies Inc., Coralville, IA, USA) was diluted in 0.2× TE buffer containing 10 ng µL^−1^ hering sperm DNA (Promega GmbH, Walldorf, Germany). For sequence information see ESI 2, S3. DNA concentration as specified by the manufacturer was confirmed using an UV-Vis spectrophotometer (Nano drop one, Thermo Fisher Scientific Inc., Waltham, MA, USA) and Qubit assay (Thermo Fisher Scientific Inc., Waltham, MA, USA). Twenty-five microliters of the complete mix was introduced into the sample inlet and emulsified using the presented cartridge. After droplet generation, the emulsion was transferred 200 µL low DNA bind tubes (Eppendorf GmbH, Wesseling, Germany) and inserted into a thermal cycler (T 100 Thermal Cycler, Bio-Rad Laboratories Inc., Hercules, CA, USA). The cycling protocol for the PCR was 5 min at 94 °C, followed by 45 cycles of 15 s at 94 °C and 60 s at 58 °C. The ddPCR assay was previously published by Schuler et al. [15].

### 4.6. ddLAMP

The ddLAMP reaction mix was prepared using 2× LyoLAMP Mastermix containing Isotherm2G DNA polymerase (myPOLS Biotec GmbH, Konstanz, Germany). A background flourophor with a concentration of 4000 nM was added for better droplet recognition of negative droplets (Alexa 647, Thermo Fisher Scientific Inc., Waltham, MA, USA). A dilution series of target DNA (62376 *Fusarium Poae* DNA, Leibniz Institute DSMZ GmbH, Braunschweig, Germany) was prepared using low DNA bind tubes (Eppendorf GmbH, Hamburg, Germany) with Ndase/Rnase free water (UltraPure 10977-035, Invitrogen Corp., Carlsbad, CA, USA). Each LAMP reaction contained final primer concentrations as follows: 1.6 μM of primers FIP and BIP, 0.4 μM of primer LB and 0.2 μM of primers F3 and B3 (for sequences see ESI 2, S3). For fluorescence detection, an intercalating dye (SYBR^®^ Green I staining reagent DNA free, AppliChem GmbH, Darmstadt, Germany) was added. Of the complete reaction mix 22.5 µL was transferred to the sample inlet and emulsified using the presented cartridge. After droplet generation, the emulsion was transferred to a 200 µL low DNA bind tubes (Eppendorf GmbH, Hamburg, Germany) and inserted into a thermal cycler (T 100 Thermal Cycler, Biorad Laboratories Inc., Hercules, CA, USA). The emulsion was incubated at 63 °C for 40 min.

### 4.7. Droplet Readout and Data Analysis

For droplet readout, 10 µL of the generated emulsion was pipetted on a commercially available counting chamber chip allowing the analysis of the droplets in a monolayer (C-Chip PK36.1, Carl Roth GmbH & Co. KG, Karlsruhe, Germany).

Bright field images were recorded with the microscope Observer Z1 (Zeiss GmbH, Jena, Germany), followed by an automated droplet diameter measurement using a custom-made ImageJ-Script (for details see ESI 2, S2).

Fluorescence images were recorded with the automatic microscope Lionheart LX (Biotek Instruments GmbH, Bad Friedrichshall, Germany) followed by automated droplet readout and counting using the software (Gen5 Image Prime, Biotek GmbH, Bad Friedrichshall, Germany).

Graphs were generated by using the graphing software Origin Pro 9 (OriginLab Corp., Northampton, MA, USA).

## 5. Conclusions

In this work, we present a centrifugal microfluidic cartridge for the generation of up to 6.65 × 10^5^ monodisperse droplets of 66 µm diameter (CV ≤ 4%). Droplet generation rates were up to 1161 droplets·s^−1^ (depending on sample viscosity), produced into standard reaction tubes by centrifugal step emulsification. With this cartridge, we were able to generate monodisperse water-in-fluorinated-oil droplets over a common range of sample viscosities directly into a standard 2 mL reaction tube. The use of fluorinated oil ensures high biocompatibility and ideal conditions for biochemical assays. The entire encapsulation process runs automatically without the need for further control. Further, no tubing, pressure pumps or any other equipment are required, which ensures ease of use even for inexperienced operators. With respect to biochemical analysis, we demonstrated that the cartridge does not compromise the assay performance of two main amplification methods for digital analysis, namely ddPCR and ddLAMP.

As the current design can easily be transferred to injection molding, the next step will allow the mass production of the cartridge making it a low-cost tool for droplet generation. Since the cartridge can be processed in a standard 2 mL reaction tube on standard laboratory equipment, no further investments are necessary.

For the current design, samples with viscosities between 1 to 4 mPa·s can be emulsified, which covers a broad range of sample types. For higher viscosities minor adjustments to the fluidic resistances of the supply channels are necessary. The same minor changes can be employed to adjust the droplet size, simply by modifying the nozzle dimensions as discussed previously by Schuler et al. [14]. By implementing design changes on the DGU and transfer structure, the cartridge could further be adapted for the use of mineral oils if needed. This flexibility makes the presented cartridge a versatile and inexpensive tool for the easy and fast production of monodisperse droplets and can be used for applications where off-chip manipulations of monodisperse droplets are required.

## Figures and Tables

**Table 1:** Measured droplet generation rate for all tested viscosity models at a fixed centrifugal acceleration of 80 g (rcf reference point see Figure 1). For detailed mixture preparation see Section 4.4. The flow rate ratios (Q_2_/Q_1_) _max_ represent the case, where both inlets are filled completely; the ratios (Q_2_/Q_1_) _min_ represent the case where both inlets are empty (for detailed calculation see ESI 2, S1). The error represents the standard deviation of two independent measurements for each tested viscosity case.

Viscosity Model (mPa·s)	Flow Rate Ratio (Q_2_/Q_1_) _max_	Flow Rate Ratio (Q_2_/Q_1_) _min_	Droplet Generation Rate (droplets·s^−1^)
1	3.56	3.40	1161 ± 28
2	1.89	1.80	918 ± 45
3	1.30	1.31	793 ± 2
4	1.00	1.10	626 ± 84

## Supplementary Materials

The following are available online: ESI 1: Stroboscopic video of the droplet generation and transfer. ESI 2: Document with additional informations (S1: Fluidic design: Detailed overview and calculation; S2: Description of the automated droplet diameter measurement with an ImageJ-Script; S3: Primer sequences ddPCR and ddLAMP; S4: Calculation of the sample concentration by Poisson statistics; S5: RAW-data (ddPCR, ddLAMP)).
