# Supplementary material for: Versatile Tool for Droplet Generation in Standard Reaction Tubes by Centrifugal Step Emulsification
Source: Molecules. 2020 Apr 21;25(8):1914. doi: 10.3390/molecules25081914 (PMC7221521; doi:10.3390/molecules25081914)
Supplement: Supplementary file 1 [file molecules-25-01914-s001.zip › ESI 2 Additional informations Schulz et al.docx]

**Electronic supplementary information (ESI) 2:**

Versatile Tool for Droplet Generation in Standard Reaction Tubes by Centrifugal Step Emulsification

Martin Schulz^1, *^, Sophia Probst^1^, Silvia Calabrese^1^, Ana R. Homann^1^, Nadine Borst^1, 2^, Marian Weiss^1^, Felix von Stetten^1, 2^, Roland Zengerle^1, 2^ and Nils Paust^1, 2, *^

^1^ Hahn-Schickard, Georges-Koehler-Allee 103, 79110 Freiburg, Germany

^2^ Laboratory for MEMS Applications, IMTEK - Department of Microsystems Engineering, University of Freiburg, Georges-Koehler-Allee 103, 79110 Freiburg, Germany

***** Correspondence: Nils.Paust@Hahn-Schickard.de; Tel.: +49-761-203-73245

S1: Fluidic design: Detailed overview and calculation

An overview of the fluidic cartridge design is displayed in Figure S1, geometry and material parameters are listed in Table S1 and S2.


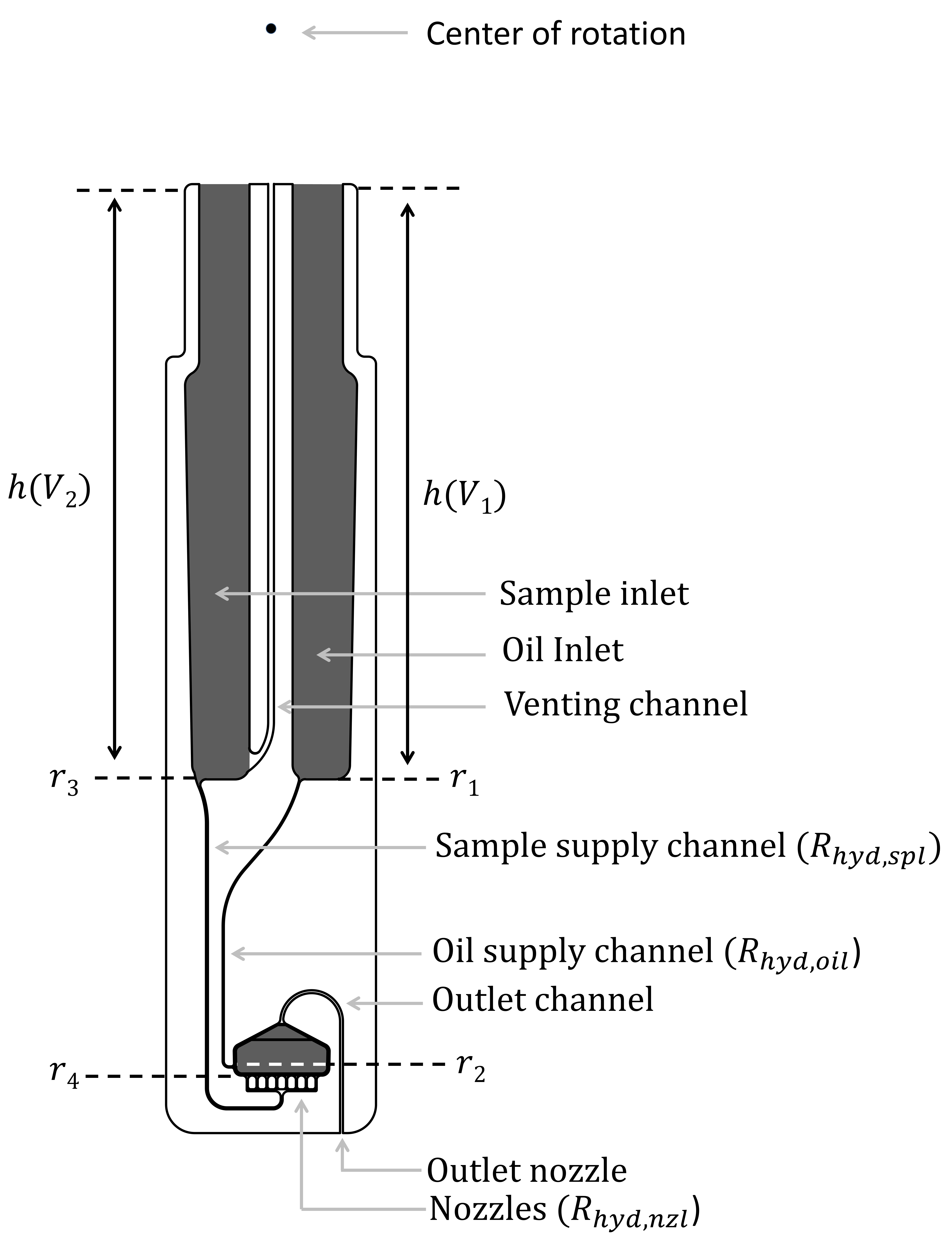


**Figure S1.** Overview of the microfluidic cartridge design with all channels and chambers marked.

**Table S1**. List of geometry and material parameters used in the microfluidic design.

| **Abbreviation** | **Description** | **Value** |
| --- | --- | --- |
| $r_{1}$ | Radial position bottom oil inlet | 47.5 mm |
| $r_{2}$ | Radial position oil supply in DGU | 57 mm |
| $r_{3}$ | Radial position bottom sample chamber | 47.5 mm |
| $r_{4}$ | Radial position nozzles | 57.37 mm |
| ${h(V)}_{1}$ | Volume height function oil | simulated |
| ${h(V)}_{2}$ | Volume height function sample | simulated |
| $d_{oil}$ | Depth oil supply channel | 37 µm |
| $w_{oil}$ | Width oil supply channel | 47 µm |
| $L_{oil}$ | Length oil supply channel | 10 mm |
| $\rho_{\mathrm{oil}}$ | Density fluorinated oil | 1614 Kg·m^-3^ |
| $\eta_{oil}$ | Dynamic viscosity fluorinated oil | 1.24 mPa·s |
| $d_{spl}$ | Depth sample supply channel | 59 µm |
| $w_{spl}$ | Width sample supply channel | 73 µm |
| $L_{spl}$ | Length sample supply channel | 12.14 mm |
| $\rho_{\mathrm{spl}}$ | Density sample | see table S2 |
| $\eta_{spl}$ | Dynamic viscosity sample | see table S2 |
| $d_{nzl}$ | Depth nozzle | 22 ± 0.69 µm |
| $w_{onzl}$ | Width nozzle | 50 ± 2.63 µm |
| $L_{nzl}$ | Length nozzle | 380 µm |
| n | Number of nozzles | 8 |
| $d_{out}$ | Depth outlet channel | 100 µm |
| $w_{out}$ | Width outlet channel | 100 µm |
| $L_{out}$ | Length outlet channel | 6.9 mm |
| $d_{vent}$ | Depth venting channel | 100 µm |
| $w_{vent}$ | Width venting channel | 200 µm |
| $L_{vent}$ | Length venting channel | 20 mm |
| V_max,oil_ | Maximum chamber volume oil inlet | 104 µl |
| V_max,sample_ | Maximum chamber volume sample inlet | 104 µl |

**Table S2**. Used glycerol-water mixtures used throughout fluidic characterization.

| **Glycerol in H_2_O (v:v)** | $\eta_{spl}$ | $\rho_{\mathrm{spl}}$ |
| --- | --- | --- |
| **(%)** | **(mPa·s)** | **(Kg·m^-3^)** |
| 0 | 1 | 1000 |
| 24 | 2 | 1067 |
| 34 | 3 | 1095 |
| 40 | 4 | 1112 |

**Flow-rate ratio calculation:**

The flow rates Q_1_ and Q_2_ were calculated by dividing the hydrostatic pressure difference ($\Delta P_{1}$; $\Delta P_{2}$) by the respective hydraulic resistance ($R_{hyd, oil}; R_{hyd, sample})$:

| $Q_{1}=\frac{\Delta P_{1}}{R_{hyd, oil}}$ | (1) |
| --- | --- |
| $Q_{2}=\frac{\Delta P_{2}}{R_{hyd,sample}}$ | (2) |

where $R_{hyd, oil}$is given by:

| $R_{hyd, oil}=\frac{12*\eta_{oil}*L_{oil}}{{d_{oil}}^{3}*w_{oil}*\left( 1- \frac{192*d_{oil}}{\pi^{5}*w_{oil}}* \left( t\mathrm{anh}\left[ \frac{\pi*w_{oil}}{2*d_{oil}} \right]+\frac{31}{32}*1.036925577-1 \right) \right)}$ | (3) |
| --- | --- |

and $R_{hyd, sample}$is given by the sum of the supply line resistance $R_{hyd, spl}$ and the nozzle resistance $R_{hyd,nzl}$:

| $R_{hyd, sample}=R_{hyd, spl}+ R_{hyd, nzl}$ | (4) |
| --- | --- |

with:

| $R_{hyd, spl}=\frac{12*\eta_{spl}*L_{spl}}{{d_{spl}}^{3}*w_{spl}*\left( 1- \frac{192*d_{spl}}{\pi^{5}*w_{spl}}* \left( t\mathrm{anh}\left[ \frac{\pi*w_{spl}}{2*d_{spl}} \right]+\frac{31}{32}*1.036925577-1 \right) \right)}$ | (5) |
| --- | --- |
| $R_{hyd, nzl}=\frac{12*\eta_{spl}*L_{nzl}}{{{n*d}_{nzl}}^{3}*w_{nzl}*\left( 1- \frac{192*d_{nzl}}{\pi^{5}*w_{nzl}}* \left( t\mathrm{anh}\left[ \frac{\pi*w_{nzl}}{2*d_{nzl}} \right]+\frac{31}{32}*1.036925577-1 \right) \right)}$ | (6) |

$\Delta P_{1}$ and $\Delta P_{2}$ are given as a function of the liquid fill level in the inlet chambers ${h\left( V \right)}_{1}$ and ${h\left( V \right)}_{2}$ and the radial positions $r_{1}$ - $r_{4}$:

| $\Delta P_{1}=\frac{\rho_{\mathrm{oil}}}{2}*\omega^{2}*(r_{2}^{2}-\left( r_{1}-\left( {h\left( V \right)}_{1} \right)^{2} \right)$ | (7) |
| --- | --- |
| $\Delta P_{2}= \frac{\rho_{spl}}{2}*\omega^{2}*(r_{4}^{2}-(r_{3}-\left( {h\left( V \right)}_{2} \right)^{2})$ | (8) |

The resulting time dependent flow rates and flow rate ratios were simulated using system-level network simulation [1]. In the main manuscript, the flow rate ratios (Q_2_/Q_1_) _max_ representing the case where both inlets are filled completely and (Q_2_/Q_1_) _min_ representing the case where both inlets are empty are listed.

S2: Description of the automated droplet diameter measurement with an ImageJ-Script

Bright field images (see Figure S2, a) were recorded with the microscope Observer Z1 (Zeiss GmbH, Germany), followed by an automated droplet diameter measurement using a custom made ImageJ-Script: First, the outlines were detected using edge detection (see Figure S2, b) followed by an automated evaluation of the area of each detected droplet (green marked, see Figure S2, c). The measured values of the droplet areas are then converted to the droplet diameter and graphed using Origin Pro 9 (OriginLab Corporation, USA). For each experiment, 1000 droplets were evaluated.

**
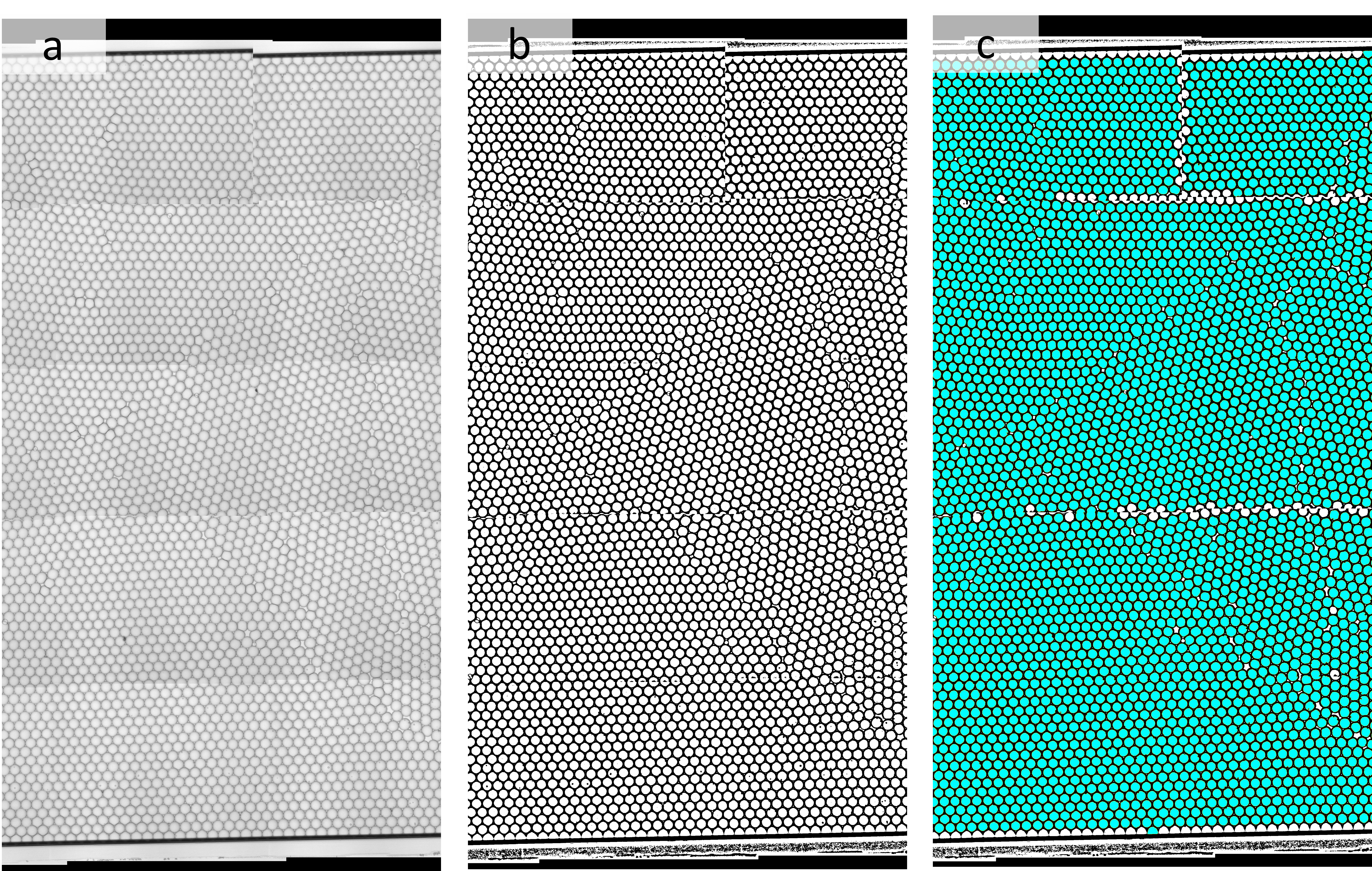
**

**Figure S2.** Example images showing the automated droplet diameter measurement using an automated ImagJ script. Original image (a). Droplet outlines (b). Detected droplets for automated droplet diameter measurement (c).

S3: Primer sequences ddPCR and ddLAMP

The following sequences were used for the ddPCR assay (see Table S3) and ddLAMP assay (see Table S4).

**Table S3**. Primer and probe sequences used in the ddPCR assay.

| Detected gene | CFTR gene |
| --- | --- |
| Forward primer | 5´-GATTATGCCTGGCACCATTAAAG |
| Reverse primer | 5´-GCATAATCAAAAAGTTTTCAC |
| Probe | 5´-VIC- AACACCAAAGATGATATT- MGB |
| gBlock-sequence | 5’-TTAAGCACAGTGGAAGAATTTCATTCTGTTCTCAGTTTTCCTGG ATTATGCCTGGCACCATTAAAGAAAATATCATCTTTGGTGTTTCCTATGATGAATATAGATACAGAAGCGTCATCAAAGCATGCCAACTAGAAGAGGTAAGAAACTATGTGAAAA |
| Source | [2] |

**Table S4**. Primer and probe sequences used in the ddLAMP assay.

| Detected gene | ESYN1 gene (enniatin synthetase) |
| --- | --- |
| FIP | 5’-GTAGCCTGCGATCGAACGTCGCCTTGGTTCAYCTRCCAG |
| BIP | 5’-CCAGGGCTGTTCGCACACTCCGTCCTCCAATTCTGCCATT |
| F3 | 5’-GCAGGTTCAACAAGCAACTC |
| B3 | 5’-TTCCCGTCGTCGGAGTAC |
| LB | 5’-GGAGAATGCAACGAAGAACGATGT |
| Source | [3] |

S4: Calculation of the sample concentration by Poisson statistics

Under the assumption that the total amount of target molecules ($m$) is distributed throughout several partitions ($n$), the probability that a partition will contain k copies of the targets can be modelled by the Poisson-distribution [4]. The expectancy value equals to the mean occupancy rate ($\lambda$), which is the ratio of the number of target (*m*) molecules to the number of partitions (*n*).

| $\lambda=\frac{m}{n}$ | (9) |
| --- | --- |
| $p_{\lambda}(k)=\frac{\lambda^{k}* e^{-\lambda}}{k!}$ | (10) |

The probability for an unallocated partition is given by:

| $p_{\lambda}\left( 0 \right)=\frac{\lambda^{0}* e^{-\lambda}}{0!}=e^{-\lambda}=N$ | (11) |
| --- | --- |

Therefore, the mean occupancy rate (λ) can be calculated from the percentage of empty partitions (N):

| $\lambda=-\ln\left( N \right)=-\ln\left( 1-\frac{k}{n} \right)$ | (12) |
| --- | --- |

By dividing the total amount of target molecules ($m$) by the reaction volume (V) the resulting concentration (c) can be calculated:

| $c=\frac{m}{V}= \frac{\lambda\cdot n}{V}$ | (13) |
| --- | --- |

S5: RAW-data (ddPCR, ddLAMP)

The RAW-data for the ddPCR and ddLAMP is listed in Table S5 and Table S6.

**Table S5**. RAW-data ddPCR.

| **Test** | **Reaction volume (V)** | **Total number of droplets (n)** | **Percentage positive droplets** | **Percentage negative droplets (N)** | **Resulting copy number (λ*n)** | **Resulting concentration** |
| --- | --- | --- | --- | --- | --- | --- |
| **[#]** | **[µl]** | **[#]** | **[%]** | **[%]** | **[cp]** | **[cp·µl^-1^]** |
| C1_1 | 25 | 1.65E+05 | 51.37 | 48.63 | 1.19E+05 | 4.76E+03 |
| C1_2 |  |  | 57.30 | 42.70 | 1.40E+05 | 5.62E+03 |
| C1_3 |  |  | 56.25 | 43.75 | 1.36E+05 | 5.46E+03 |
| C2_1 | 25 | 1.65E+05 | 10.23 | 89.77 | 1.78E+04 | 7.12E+02 |
| C2_2 |  |  | 10.54 | 89.46 | 1.84E+04 | 7.35E+02 |
| C2_3 |  |  | 8.99 | 91.01 | 1.55E+04 | 6.21E+02 |
| C3_1 | 25 | 1.65E+05 | 0.63 | 99.37 | 1.04E+03 | 4.18E+01 |
| C3_2 |  |  | 0.59 | 99.41 | 9.78E+02 | 3.91E+01 |
| C3_3 |  |  | 0.59 | 99.41 | 9.83E+02 | 3.93E+01 |

**Table S5**. RAW-data ddLAMP.

| **Test** | **Reaction volume (V)** | **Total number of droplets (n)** | **Percentage positive droplets** | **Percentage negative droplets (N)** | **Resulting copy number (λ*n)** | **Resulting concentration** |
| --- | --- | --- | --- | --- | --- | --- |
| **[#]** | **[µl]** | **[#]** | **[%]** | **[%]** | **[cp]** | **[cp·µl^-1^]** |
| C1_1 | 22.5 | 1.50E+05 | 4.85 | 95.15 | 7.45E+03 | 3.31E+02 |
| C1_2 |  |  | 3.58 | 96.42 | 5.47E+03 | 2.43E+02 |
| C1_3 |  |  | 5.03 | 94.97 | 7.73E+03 | 3.44E+02 |
| C2_1 | 22.5 | 1.50E+05 | 0.61 | 99.39 | 9.20E+02 | 4.09E+01 |
| C2_2 |  |  | 0.47 | 99.53 | 7.03E+02 | 3.12E+01 |
| C2_3 |  |  | 0.88 | 99.12 | 1.33E+03 | 5.90E+01 |
| C3_1 | 22.5 | 1.50E+05 | 0.09 | 99.91 | 1.32E+02 | 5.88E+00 |
| C3_2 |  |  | 0.04 | 99.96 | 5.82E+01 | 2.59E+00 |
| C3_3 |  |  | 0.08 | 99.92 | 1.25E+02 | 5.58E+00 |

References

1. Schwarz, I.; Zehnle, S.; Hutzenlaub, T.; Zengerle, R.; Paust, N. (2016): System-level network simulation for robust centrifugal-microfluidic lab-on-a-chip systems. In: Lab Chip 16 (10), S. 1873–1885. DOI: 10.1039/c5lc01525a.
2. Schuler, F.; Trotter, M.; Geltman, M.; Schwemmer, F.; Wadle, S.; Domínguez-Garrido, E.; López, M.; Cervera-Acedo, C.; Santibáñez, P.; Stetten, F. von; et al. Digital droplet PCR on disk. Lab Chip 2016, 16, 208–216, doi:10.1039/c5lc01068c.
3. Chinese patent application CN106893763A “LAMP detection primer combination of fusarium poae and LAMP detection kit and LAMP method of fusarium poae” Applicant: Institute of plant protection, Chinese academy of agricultural sciences.
4. Basu, Amar S. (2017): Digital Assays Part I: Partitioning Statistics and Digital PCR. In: *SLAS technology* 22 (4), S. 369–386. DOI: 10.1177/2472630317705680.
